# Using an equity-based framework for evaluating publicly funded health insurance programmes as an instrument of UHC in Chhattisgarh State, India

**DOI:** 10.1186/s12961-020-00555-3

**Published:** 2020-05-25

**Authors:** Sulakshana Nandi, Helen Schneider

**Affiliations:** 1 School of Public Health, University of the Western Cape, Bellville, South Africa; 2 Public Health Resource Network, 29, New Panchsheel Nagar, Raipur, Chhattisgarh 492001 India; 3 School of Public Health, UWC/MRC Health Services to Systems Unit, University of the Western Cape, Bellville, South Africa

**Keywords:** Universal health coverage, Publicly funded health insurance, Equity, Access, HPSR, India

## Abstract

**Background:**

Universal health coverage (UHC) has provided the impetus for the introduction of publicly funded health insurance (PFHI) schemes in the mixed health systems of India and many other low- and middle-income countries. There is a need for a holistic understanding of the pathways of impact of PFHI schemes, including their role in promoting equity of access.

**Methods:**

This paper applies an equity-oriented evaluation framework to assess the impacts of PFHI schemes in Chhattisgarh State by synthesising literature from various sources and highlighting knowledge gaps. Data were collected from an extensive review of publications on PFHI schemes in Chhattisgarh since 2009, including empirical studies from the first author’s PhD and grey literature such as programme evaluation reports, media articles and civil society campaign documents. The framework was constructed using concepts and frameworks from the health policy and systems research literature on UHC, access and health system building blocks, and is underpinned by the values of equity, human rights and the right to health.

**Results:**

The analysis finds that evidence of equitable enrolment in Chhattisgarh’s PFHI scheme may mask many other inequities. Firstly, equitable enrolment does not automatically lead to the acceptability of the scheme for the poor or to equity in utilisation. Utilisation, especially in the private sector, is skewed towards the areas that have the least health and social need. Secondly, related to this, resource allocation patterns under PFHI deepen the ‘infrastructure inequality trap’, with resources being effectively transferred from tribal and vulnerable to ‘better-off’ areas and from the public to the private sector. Thirdly, PFHI fails in its fundamental objective of effective financial protection. Technological innovations, such as the biometric smart card and billing systems, have not provided the necessary safeguards nor led to greater accountability.

**Conclusion:**

The study shows that development of PFHI schemes, within the context of wider neoliberal policies promoting private sector provisioning, has negative consequences for health equity and access. More research is needed on key knowledge gaps related to the impact of PFHI schemes on health systems. An over-reliance on and rapid expansion of PFHI schemes in India is unlikely to achieve UHC.

## Introduction

India has a mixed health system, with a large public sector that is underfunded and fraught with numerous challenges, and a rapidly growing, unregulated and heterogeneous private sector [1, 2]. Over the last decade and a half, there have been two major strands of health sector reform in India’s mixed health system. The first, the National Rural Health Mission (since renamed the National Health Mission) was launched in 2005 and emphasises strengthening public health systems to provide effective healthcare and improve “*access, equity, quality, accountability and effectiveness of public health services*” [3]. The second strand, initiated a few years after the National Rural Health Mission was launched, targets catastrophic health expenditure by the poor in a variety of state-level publicly funded health insurance (PFHI) schemes in states such as Karnataka, Andhra Pradesh and Tamil Nadu [4]. The Rashtriya Swasthya Bima Yojana (RSBY) or the National Health Insurance Scheme, launched by the Ministry of Labour in 2007, was the first national-level PFHI scheme for the unorganised sector, providing insurance cover of INR 30,000 (US$ 424) to Below Poverty Line households for hospitalisation. RSBY sought to draw extensively on private health sector providers. In 2018, the PFHI scheme was further expanded through Prime Minister Jan Arogya Yojana (PMJAY) under Ayushman Bharat, to an annual coverage of INR 500,000 (US$ 7072) per family [5]. This scheme is expected to cover 100 million families and 500 million people for hospitalisation costs, corresponding to around 37% of India’s population [5].

One of the core rationales advanced for the introduction of PFHI schemes in India has been the achievement of universal health coverage (UHC) [6, 7]. UHC is a globally advocated concept that aims to ensure “*that everyone within a country can access the health services they need, which should be of sufficient quality to be effective, and providing all with financial protection from the costs of using health services*” [8]. Initially, apart from financial barriers to access, equity and access were not explicitly part of the discourse on UHC [9–12]. Equity emerged more strongly in subsequent articulations, and there is now global consensus that any country moving towards UHC has to ensure equity as a primary goal [12–15].

In India, inequity is related to socioeconomic and political status, caste, class, geography and gender differences, amongst others, resulting in inequitable health outcomes, health service utilisation and access to healthcare [16–18]. These dimensions also converge and intersect, exacerbating individual inequities [19, 20]. Health systems play an important role in either deepening or addressing wider social inequity [21–23].

As a major current reform in India, it is important to evaluate PFHI schemes and, in particular, their impacts on equity of access. So far, studies on PFHI schemes in India and in other low- and middle-income countries (LMICs) have focused more on enrolment, utilisation and financial protection [24, 25] and less on understanding the perceptions and experiences of people who have tried to use such schemes [26, 27], or on the pathways of impact of PFHI through the overall health system and their relationship to equity; there have been some attempts to analyse the policy-making process [28, 29].

Studies of PFHI schemes in other LMICs (Indonesia, Ghana, Nigeria, Vietnam, Philippines, Rwanda, Kenya and Mexico) have commonly found lower enrolment among the poor, with differences based on rural–urban divide and education [30–34]. However, where schemes are specifically designed to enrol the more vulnerable, better coverage of the poor and of rural populations is achieved [25, 32].

The evidence on utilisation under PFHI schemes is mixed [34]. Some studies show increases in financial protection and healthcare utilisation with enrolment [35, 36], in some instances with pro-poor patterns [37]. Others have found that the expansion of health insurance did not necessarily lead to increased financial protection indicators or a decrease in out-of-pocket (OOP) expenditure [38, 39]. The poor and people in remote areas with poorly staffed facilities tend to have less utilisation and financial protection [34, 40–43].

In India, lower enrolments have been reported in remote rural areas and poorer districts, among socioeconomically vulnerable communities such as tribal communities, in female-headed households and in the poorer quintiles [44–48]. While the overall enrolment of women in RSBY has been increasing and is equal to that of men [49], enrolment seems to have become an additional barrier for women to access health services [50].

Similar to the international experience, the impact of PFHI schemes on hospitalisation has been mixed in India. Exclusion during enrolment subsequently translated to lower utilisation by the excluded groups [47]. Studies highlight the inequitable distribution of empanelled hospitals, especially of hospitals in the private sector, leading to inequitable access [51, 52]. The utilisation of the public sector is higher for the poor and vulnerable groups even with insurance [46]. The proportion of women being hospitalised under the PFHI schemes is higher than that of men but they have also been more vulnerable to provider-induced demand [50, 53–55].

The majority of the studies in India have found that significant levels of OOP expenditure continue despite insurance coverage, and that PFHI schemes have failed to protect against catastrophic health expenditure [24, 46, 56–58], especially the poor [59]. OOP expenditure is higher when utilising private facilities due to impermissible co-payments [44, 59, 60]. Practices by the private sector, of converting outpatient to inpatient care, ‘cherry picking’ of more profitable packages, and provision of a selective and narrow set of services have been documented [61, 62]. Lack of transparency and access to data, information and grievance redress mechanisms have also been highlighted as problems under PFHI schemes in India [48, 51, 63].

With the Indian government expanding health insurance through the PMJAY, stepping up research on PFHI is of high priority. This is especially important in the light of concerns related to the impact on financial protection, dynamics of the private and public provisioning, impact on health priorities, health budgets and health equity and, ultimately, the ethical basis of such policies [5, 56, 64, 65]. In this context, there is a need for a holistic understanding of the pathways of impact of such schemes that take into account equity and access. Such an analysis is vital to understanding whether the policy push towards PFHI schemes is achieving the stated policy goal of achieving UHC.

### Aims

This paper applies an equity-based framework to holistically assess the pathways of impact of PFHI schemes in Chhattisgarh State, from values and objectives, to design, enrolment, health system effects, equity of access and, ultimately, people and populations. It proposes a conceptual framework for evaluating India’s PFHI schemes as instruments of UHC reform and then, using the framework, the paper synthesises available evidence on the design, implementation and equity impacts of the PFHI schemes from Chhattisgarh, highlighting the current state of and gaps in knowledge.

### Conceptual framework

**Fig. 1:**
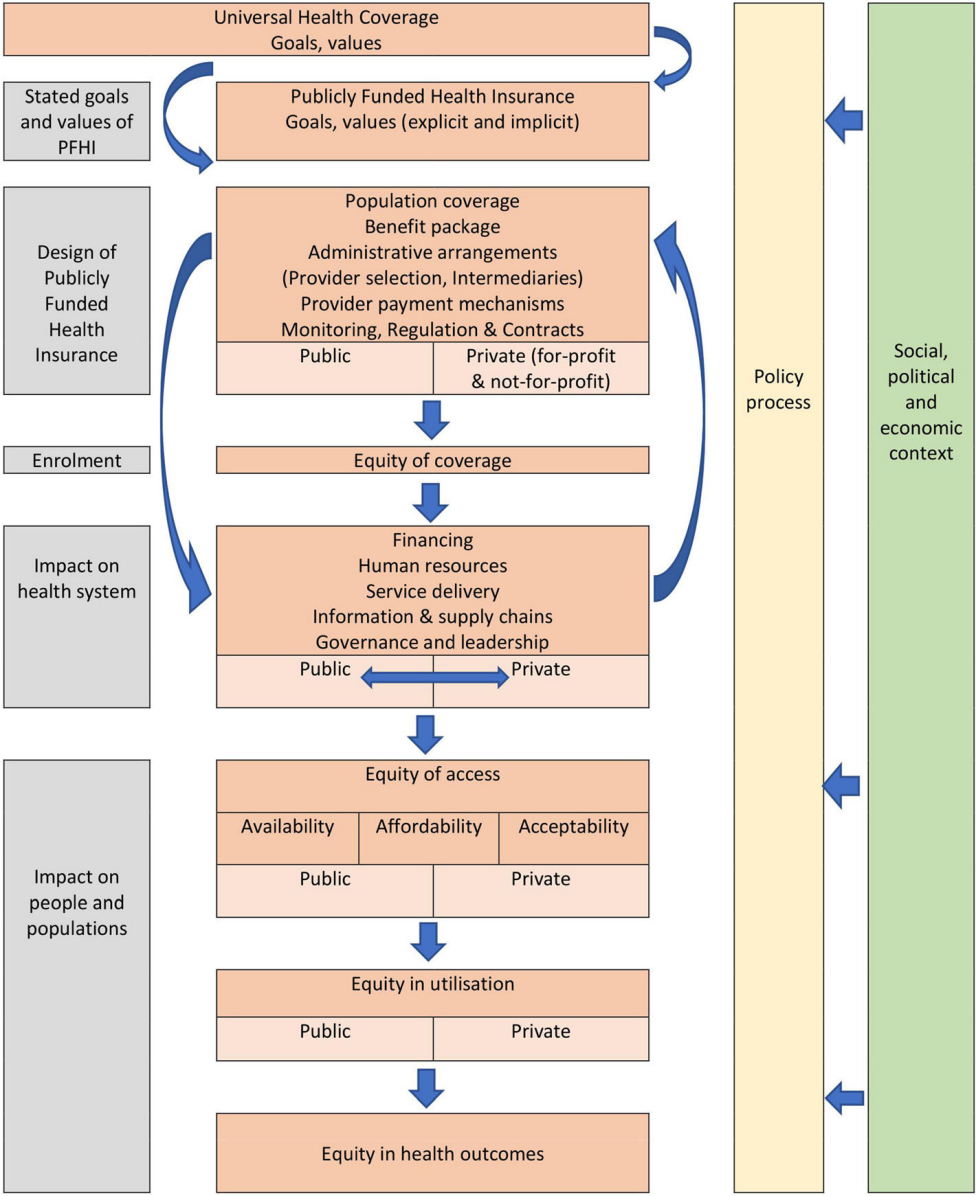
A framework for assessing pathways of impact on equity of access in publicly funded health insurance programmes for universal health coverage in low- and middle-income countries

The conceptual framework for assessing pathways of impact on equity of access in PFHI schemes for UHC in LMICs is presented diagrammatically in Fig. 1.

The framework has been constructed using concepts and frameworks from the health policy and systems (HPSR) literature on UHC, access, health system building blocks, healthcare provisioning in mixed health systems and policy analysis, and is underpinned by the values of human rights, including the right to health and equity. The specific inputs into the framework are summarised in Table 1, along with the research questions that may be explored under each concept. The framework seeks to be relevant to researchers, policy-makers, journalists and anyone interested in studying or understanding PFHI schemes and their implications for the health system, people and populations.

**Table 1:** Inputs into the framework and research questions

Category	Key concepts, frameworks and principles	Research questions	References
UHC and its critiques and alignment of the PFHI scheme with UHC objectives	- Three core UHC goals of financial protection, coverage of population and of services In addition: - Importance of equity within the above - Focus on appropriateness and quality of services - Acknowledgement of: • The right to health for all • Social determinants of health • Strengthening of public health systems • Promotion of health as a social good and not a commodity - Emphasis on universal health systems rather than UHC Value base: - Right to health - Human rights - Equity of access - Equity of outcome	Do the objectives of the PFHI scheme take into account financial protection, coverage of population and of services, and have equity considerations? Do the objectives refer to the health system as a whole? Is there foregrounding of equity in monitoring and evaluation of the PFHI scheme? Does it cover a limited package of health services or does it talk about UHC and systems? Are appropriateness and quality of services made explicit and addressed? Is the scheme aligned with the idea of health being a fundamental right? Does the scheme promote health as a social good and a right or as a commodity? Are the social determinants of health and structural drivers of health inequity acknowledged and addressed within the scheme? Has the scheme led to equitable access? Has the scheme led to equitable outcomes?	[9, 10, 12, 66–77]
Design of PFHI	Key design issues include: - Population coverage - Benefit package - Administrative arrangements - Provider selection, intermediaries - Provider payment mechanisms - Monitoring, regulation and contracts - Transparency, access to information and accountability	Who is covered under the PFHI scheme and what does it mean in terms of equity of coverage? What kinds of services are covered? What are the administrative and implementation arrangements? Who pays? How is the payment of premiums organised? How will the provider be paid? How is the provider selected? Are both the private and the public sectors to provide services under the scheme? What are the systems for monitoring, regulation and contracts? To what extent are equity considerations central to the design? Is information on all aspects of the scheme publicly available? What systems of public accountability are in place?	[78]
Impact on health system	Building blocks: - Financing - Human resources - Service delivery arrangements - Information - Equipment and supply chains - Governance and leadership Importance of studying: - Interactions between the building blocks - Both hardware (building blocks) and software (values, relationships) - Health systems as complex adaptive systems	What has been the impact of the PFHI scheme on financing, service delivery, human resources and supply chains? Has it been different for the private and public sectors? Have there been any equity implications of the above, especially with regards to changing resource allocations and use of earmarked funds for vulnerable groups? What have been the interactions among the building blocks of health systems, both among the system’s hardware (organisational, policy, legal and financing frameworks) and software (norms, traditions, values, roles and relationships)? How effective are the systems for monitoring, transparency and accountability?	[79–83]
Impact on people and populations	- Dimensions of access: availability, affordability and acceptability - Three dimensions of access interact with each other to create the opportunity for utilisation and the possibility of improved health outcomes - Access needs to be defined in relation to health needs - Acceptability as under-studied, including empowerment, agency, capacities to ‘navigate and negotiate’ and understanding interactions, perspectives and contexts	Is the availability of health facilities and health services, which includes quality (‘effective coverage’) and appropriateness of services, equitable? What has been the out-of-pocket expenditure, including catastrophic expenditure, incurred by the patient/family for healthcare or while utilising insurance? Is it higher or lower for vulnerable people/groups? Is a conducive service delivery environment being provided under the PFHI scheme with mechanisms for negotiating and navigating the system, and providing information, transparency, accountability and agency to patients? What is the nature of the grievance redressal system? Is it effective, especially for the poor and more vulnerable groups? How do the three dimensions create the opportunity for utilisation and what is the implication for equity?	[13, 84–88]
Public/private sector interactions	- In health systems and schemes that have a public–private mix in provision of health services, it is critical to examine the role of each sector separately in ensuring access and equity and furthering the objectives of UHC	Has the impact of the PFHI scheme been different on the private and public sectors? Has the introduction of a PFHI scheme altered the healthcare provision practice of private and public sector providers? What have been the implications for equity in access?	[2, 89–92]
Policy process	- Policy change as a political process - Process of agenda-setting, decision-making, formulation, implementation and evaluation - Role of context, actors, interests, ideas, power relations and institutions - Located within the political economy of development	Who are the key actors in the policy process? How does power play out amongst these actors? What is the impact of social structures and power differentials on the functioning of the scheme? What is the nature of the political economy of healthcare within which this scheme has been introduced and how does that influence equity?	[93–95]

*PFHI* publicly funded health insurance, *UHC* universal health coverage

## Methods

As a multi-disciplinary field, HPSR draws on a wide variety of research designs, data collection methods (both qualitative and quantitative) and sources of data, pragmatically identifying the research methodologies best able to answer questions generated by real-world problems [93]. In doing so, HPSR frequently adopts research approaches outside mainstream biomedical approaches in order to better understand interaction, perspectives and contexts [13] or issues of power and politics [96].

This paper similarly draws on a range of data sources in applying the above conceptual framework to an analysis of PFHI schemes in Chhattisgarh state. The sources of data included an extensive review of publications on PFHI schemes in Chhattisgarh after 2009, including research and media articles by the first author and empirical studies from her PhD, and grey literature such as programme evaluation reports, media reports, and civil society campaign and advocacy documents.

The first author has, since 2010, kept a database on publications related to PFHI schemes in Chhattisgarh. This database was re-checked and updated through additional searches of published (Medline database) and grey literature. For the grey literature, website searches were conducted of state and central government, media houses, agencies such the World Bank, WHO and Deutsche Gesellschaft für Internationale Zusammenarbeit. The search terms were a combination of Chhattisgarh, health insurance, UHC, RSBY, Mukhyamantri Swasthya Bima Yojana (MSBY) and PMJAY. The time period for the search was from 2009, the year RSBY was launched. All the articles, reports and other publications in the updated database were included in the analysis. Using a framework analysis approach, SN read the available sources and identified relevant data for each element of the conceptual framework.

As this study is based on secondary data analysis, consent procedures were not required. Ethics approval for the overall PhD research of which this study forms one component, was obtained from the University of the Western Cape, South Africa where the first author (SN) is registered.

## Results

### Applying the framework to Chhattisgarh state

Chhattisgarh was carved out as a separate state from Madhya Pradesh in 2000, and has a population of about 25 million people, with 77% of families living in rural areas [97]. Scheduled Tribes constitute 31% of the population while 13% are from the Scheduled Caste category [97]. Forested areas cover 41% of the State’s total geographical area [98]. Although Chhattisgarh has seen a significant improvement in health indicators since 2000 [18, 99], it still lags behind national averages [100].

One of the first states to launch RSBY in 2009, Chhattisgarh expanded PFHI scheme to all families of the state in 2012 through the MSBY. RSBY covered people living below the poverty line and MSBY covered those above the poverty line. These two schemes together made up a universal scheme. Since September 2018, RSBY has been subsumed under PMJAY.

In the following section, the equity-based framework is applied to the case of the universal PFHI scheme (RSBY/MSBY) in Chhattisgarh, presenting the pathways of impact from values and objectives, to design, implementation, and impact. The results relating to the impact of PFHI schemes on the health system and people and populations are disaggregated by private and public sector. Further, results are presented taking into account the overarching dimensions of the policy process and social, political and economic context (Fig. 1). It must be recognised that elements of the framework are overlapping. For instance, a study of financing and affordability can speak to both the impact on health system and on people. For clarity and understanding of the framework, the same data are sometimes reported more than once.

#### Alignment of goals of PFHI in Chhattisgarh with UHC

The overall framing of the PFHI scheme in Chhattisgarh has followed that of the national scheme that has alignment with globally articulated UHC goals [28, 29], though it has not been studied separately as an instance of UHC. While RSBY covered the poor, the stated objectives of both RSBY and MSBY are similar and reflect the UHC principles and goals, bringing together ideas of universal insurance coverage, financial protection, equity, access and private sector involvement. This is seen in the articulation by government on its website that states the following: “*Health insurance protects against the cost of illness, mobilizes funds for health services, increases the efficiency of mobilization of funds and provision for health services, and achieves certain equity objectives*” [101]. The website also calls for “*involvement of community in rural health care for increasing accountability*”, ensuring “*choice to patients among multiple service providers*”, encouraging public–private partnerships, and improving mortality and morbidity rates [101]. The equity dimension is operationalised through universalisation of the PFHI scheme. However, while the dimensions of financial protection and equity are clearly visible in the stated objectives, it lacks considerations regarding the health system as a whole. There is much emphasis on engaging the private sector and engaging in public–private partnerships [101]. Analyses of the genesis of RSBY nationally have highlighted that the PFHI scheme was seen as an ‘investment’ in worker productivity and influenced by considerations of human capital development, efficiency and productivity rather than ‘needs’ or ‘rights’, and this led to a narrow and selective scheme design [28, 29]. Moreover, PFHI schemes have been promoted as a ‘business’ and market-oriented model prioritising a for-profit motive [28].

#### Design of PFHI in Chhattisgarh

All families living in the state are eligible for enrolling in the scheme, creating a single risk pool. Both MSBY and RSBY cover a maximum of five family members and require an annual enrolment process, following which an annual premium is paid by the government on behalf of all families to the insurance company. There is active enrolment through enrolment camps in villages and health facilities, during which the family is issued a biometric smart card [102]. The smart card is intended to be used for ‘cashless’ hospitalisation at empanelled private and public hospitals. In addition, as part of regular government policy, people are able to utilise health services, including hospitalisation, at all public facilities free of cost or at a low cost without using the insurance smart card. In 2017, the state expanded the annual insurance cover from INR 30,000 (US$ 424) to INR 50,000 (US$ 707).

As elsewhere in India and in many LMICs, PFHI schemes in Chhattisgarh cover a limited package of services, mostly provided by the private sector. The benefit package under PFHI schemes in Chhattisgarh includes hospitalisation and a few non-hospitalised packages such as dentistry and antenatal care. PFHI schemes in India have been critiqued for mostly covering hospitalisation services, and some have argued for its expansion to include out-patient services [56]. However, others have highlighted the greater potential risk of fraud in out-patient care [103].

In India, there are two models of purchaser organisation in the PFHI schemes. One kind of purchaser organisation is a trust which is an autonomous organisation set up by the state government to empanel hospitals and pay the claims. The second involves hiring an insurance company to handle third-party payments based on the premium paid. In Chhattisgarh the purchasing arrangement is through an insurance company, selected by government in a bidding process. The insurance company, in turn, appoints a third-party agency (TPA) to process the claims. The TPA is also responsible for enrolling all households.

The provider payment mechanism is a mix of per case, per procedure and per day rates. Contracts between the state, the insurance company and the hospitals lay down the guidelines and conditions for providing services under the scheme. The providers are required to provide ‘cashless’ treatment on the basis of the pre-determined package rates and are prohibited from taking any other charges from patients. The use of biometric smart cards and a helpline number are seen as the tools for monitoring transactions and ensuring grievance redressal. Oversight of the scheme is with the State Nodal Agency, under the Department of Health and Family Welfare.

While in the initial few years, utilisation of the scheme was low leading to profits for the insurance company [104], the claim amounts subsequently exceeded the total premium paid. As a consequence of increasing claim amounts every year, the insurance premiums in Chhattisgarh have also increased above inflation rates. For instance, premiums more than doubled from INR 314 (US$ 4.4) in 2012–2013 to INR 732 (US$ 10) per family in 2016–2017 for an annual cover of INR 30,000 (US$ 424) per family [60]. Currently, for a cover of INR 50,000 (US$ 707) under PMJAY in Chhattisgarh, the annual premium is INR 1100 (US$ 15.6) per family [60].

Chhattisgarh has one of the highest enrolment rates in the country [18]. Recent programme data shows enrolment to be 80% of families and 60% of individuals [60]. In terms of equity of coverage, enrolment has been found to be equitable across gender, social groups and economic categories [105, 106] and highest in the most vulnerable districts [106]. However, smaller primary studies show that enrolment percentages among some of the most vulnerable communities are much lower. For instance, in a 2016 survey among the Baiga Particularly Vulnerable Tribal Group, 38% of families were found to be enrolled [107]. An earlier study documented instances of the TPA bypassing ‘remote’ and inaccessible villages in enrolment drives [61].

#### Impact on health system

Research is lacking on the impact of PFHI schemes on the health system. The section below describes ways to assess the impact of PFHI schemes on the health system, using available literature from Chhattisgarh.

##### Financing

Increases in insurance premiums have impacted on resource allocation in health sector budgets. The share of RSBY/MSBY in the health budget has doubled in the last 3 years, from 6.6% in 2015–2016 to 13% of the total health budget in 2018–2019, while the budgets for many other health programmes have been stagnant or reduced [108].

Programme data on claims and their amounts by region, social group (ST and SC), income and gender shows that the most vulnerable districts (mainly areas with higher ST and SC population) receive the least funds [104, 106]. Yet, a significant proportion of the PFHI scheme funds are sourced from the Tribal sub-plan (39% in 2018–2019) and other budgets meant for the welfare of STs and SCs [60].

Analysis of flows of funding to public and private sectors show that the private sector receives a much higher proportion of the claims amount than the public sector. In 2015–2016, the private sector made 75% of the claims and received 83% of the claims funds [106], a pattern that has remained the same since the beginning of the scheme [104].

##### Service delivery

Currently, 609 government hospitals and 588 private hospitals are empanelled [101]. Patterns of utilisation of PFHI in the state show that 87% of the claim amount goes for only the top 5% of the listed procedures, among which dental, deliveries, cataract and medical management of diarrhoea/fever are used most commonly [109]. Media reports suggest that this pattern has continued under PMJAY as well [110]. On the other hand, when conditions like multidrug-resistant tuberculosis were introduced under PFHI, the participation of the private sector was reported to be extremely low [111]. This reflects the practice of ‘cherry picking’ and the provision of narrow and selective services in the private sector documented in formal research [61, 109].

Instances of moral hazard and provider-induced demand, typically associated with insurance schemes, have also been documented, including cases of unnecessary hysterectomies by the private sector under RSBY [52, 112, 113]. One media report indicated that, over a period of just 8 months in 2012, private hospitals submitted claims for 1800 hysterectomies in Chhattisgarh, many of which were later deemed unnecessary [113].

##### Human resources

Research analysing the impact of PFHI schemes on human resource dynamics and labour markets in the private and public sectors along with their implications for equity has yet to be conducted. Observations and anecdotal evidence suggest that PFHI schemes have increased opportunities for dual practice by public sector providers and collusive behaviour in terms of referrals from the public to the private sector [114].

##### Equipment and supply chains

Similarly, there have not been any studies on the impact of PFHI schemes on pharmaceutical and medical device markets, and the effects on their supply and availability in the public sector. Specific research questions that could guide such an enquiry are listed in Table 1.

##### Practices of governance and leadership

The oversight and guidance functions of the health system have also not been explicitly examined in Chhattisgarh’s PFHI schemes. A qualitative study by the authors revealed a breakdown of mechanisms for regulation and monitoring, specifically in the private sector, that is reflective of the overall lack of regulation of healthcare in the state and country [115]. The biometric smart card did not function as a guarantee of ‘cashless’ transactions as intended and, instead, at times served to extract additional payments from the patients. Grievance redressal mechanisms, when used, failed to provide relief [115]. Another study documented conflicts of interest of public officials who were in charge of decisions related to PFHI policy (for instance, what services to include and their pricing) and monitoring of the private sector, who were part-time private providers themselves [60].

Media reports, documentation by civil society and participant observation by the first author, have provided insights into the consequences of persistent governance failures under the PFHI schemes. These include lack of action on co-payments in the for-profit private sector or forcing patients to buy medicines even though they are covered by the insurance packages [114, 116–118].

##### Information

Advances in information technology have been seen as the mainstay of information systems under PFHI schemes. Mechanisms such as the biometric smart card and information technology-based billing systems are supposed to enable real-time data, patient access to information and transparency. However, studies have found that patients are often not told about the amounts deducted from their insurance smart card nor given receipts [104, 115, 119]. Some rural area facilities faced problems in utilising the scheme due to the lack of regular internet connectivity [61]. Private hospitals have reported being more able to handle the technological requirements while government hospitals faced problems, resulting in higher rejection of claims in these facilities [61].

#### Impact on people and populations

As proposed in the framework, the impact of PFHI on people and populations can be assessed through constructs of access (availability, affordability and acceptability), resulting in utilisation and ultimately in health outcomes. Each of these dimensions is approached from an equity perspective, and public and private sectors are considered separately. These elements and dimensions have been studied extensively by the authors themselves.

Analysis of equity in availability of hospital services under PFHI schemes in Chhattisgarh showed that, while government hospitals are relatively evenly distributed, most of the private hospitals are concentrated in only a few cities and their distribution skewed towards the districts having least vulnerability [106, 119]. Another study showed that the more vulnerable groups, such as women, people living in rural areas, Scheduled Tribes and poorer groups, were more likely to utilise the public than the private sector for hospitalisation [105].

Availability of hospital services also includes the quality and appropriateness of care (the concept of effective coverage) though this aspect has been less studied [120]. As highlighted in the previous section, there are indications that the PFHI schemes have enabled new forms of provider-induced demand and promoted patterns of selective provisioning [61, 109].

The authors’ study of affordability based on household survey data revealed that, of those who were insured and used private hospitals, only 5% received free services, while, of those who were insured and used public hospitals, 34% did not incur any OOP expenditures [105]. Of the insured who incurred OOP expenditures, the median expenditure in the private sector (INR 10,000 or US$ 141) was eight times more than that in the public sector (INR 1200 or US$ 17) [105]. While those covered with insurance were less likely to incur OOP expenditure, women and those going to private hospitals were significantly more likely to incur OOP expenditure [105]. Of households with at least one case of hospitalisation, 35.5% incurred catastrophic health expenditure (>10% monthly household consumption expenditure) [105]. The main reason for continuing OOP expenditure has been impermissible co-payments that are charged by the hospitals from patients who use PFHI schemes [109, 115].

Primary studies have complemented the analysis of routine household surveys, finding OOP expenditure continuing among the urban poor and extremely low utilisation of the scheme by Particularly Vulnerable Tribal Groups [104, 107, 115, 119].

Studies assessing the level and nature of information provided to the beneficiaries have found that families were not provided the list of empanelled hospitals to choose from [104, 119, 121]. The qualitative study by the authors among families who had incurred high OOP expenditure while utilising the PFHI scheme in the private sector found that patients and their families exercised their agency to the extent they could but were rendered helpless and powerless when hospitals demanded extra payments [115]. The smart card, instead of being a vehicle to ‘empower’ the patient and enable ‘cashless’ services, was perceived as an opportunity to extract additional money [115]. The inability of the PFHI schemes to ensure financial protection in these instances arose from a combination of prevailing social norms, such as care as a market transaction rather than a right, wider cultural acceptance of illegal informal healthcare payments, power asymmetries between patients and providers, and the failures of regulatory mechanisms and oversight [115].

Utilisation of health services flows from the three dimensions of access and their interaction. In Chhattisgarh, utilisation of PFHI schemes follows the pattern of inequitable availability of hospital services across districts [106]. The most vulnerable districts had 3.5 times lower claims (per 100,000 enrolled) than low-vulnerability districts, with claim amounts following similar patterns [106]. No studies have been done as yet on the impact of PFHI schemes on health outcomes in Chhattisgarh.

#### Policy process

This section deals with the policy context, actors and processes under PFHI schemes. Studies have analysed the evolution of PFHI in India [28, 29] but not specifically in Chhattisgarh. These studies have given rise to a number of interpretations of the policy process, which in Chhattisgarh and elsewhere is still unfolding.

Chhattisgarh implemented RSBY as part of the national scheme but expanded it to universal coverage with its own funds through the Chief Minister’s Health Insurance Scheme or MSBY, that included the non-poor. Chhattisgarh is known for leading innovations in healthcare and in other areas of public policy such as initiating a large Community Health Worker Programme [122], a 3-year medical diploma course to address the shortages of health practitioners in rural areas [123] and a near-universal public distribution system (PDS) providing subsidised grain [124].

The policy direction of a universal PFHI scheme was possibly prompted by the ruling party’s previous political success with the much applauded near-universal PDS. In the case of the PDS, the state had similarly elected to expand coverage to families not covered by the national food scheme, through a new scheme called the Chief Minister’s Food Relief Scheme or MKSY [124]. The expansion of PFHI scheme in the state occurred in 2012, just before the state elections in 2013.

The actors involved in policy-making and in advocating for PFHI at the national level have also influenced developments in Chhattisgarh State to an extent. The strong proponents of PFHI include the NITI Aayog (a policy think tank of the Indian government), the National Health Authority, international agencies such as the World Bank, Deutsche Gesellschaft für Internationale Zusammenarbeit and the Asian Development Bank, UN agencies (WHO, International Labour Organization), and philanthropic foundations such as Bill & Melinda Gates Foundation. While the Health Ministry remains involved both at the national and the state levels, an autonomous institution, the National Health Authority, has been formed to implement PMJAY. Groups representing private healthcare providers such as the Indian Medical Association have been vocal in demanding higher package rates and lesser regulation. In 2013, the Indian Medical Association staged a strike for close to 3 months in the main cities of Chhattisgarh, suspending all services under PFHI, to pressurise the government to increase the package rate [52]. PFHI schemes remain heavily contested in Chhattisgarh and therefore the newly elected state government has decided to review the PFHI schemes in the state [125].

## Discussion

Studies on PFHI schemes in India and elsewhere have been limited to one or another element and have not studied impacts as a whole [24, 34, 126]. There has been a dearth of studies on assessing the impact of such schemes for UHC, on the health system and the population [10]. Studies on dimensions of access (availability, affordability and acceptability) in PFHI schemes have mostly studied financial protection or the affordability dimension, with less focus on the availability and acceptability dimensions [26, 27, 127]. PFHI schemes as instruments of UHC also need to be assessed in terms of their contribution to equity, human rights, quality and appropriateness of health services, strengthening of public health systems and promotion of health as a social good [9, 12, 71–75, 128]. Most studies on PFHI schemes have failed to examine public and private sectors separately [34], although recent studies in India have started to examine financial protection by each sector [24, 46].

By formulating an overall conceptual framework for PFHI schemes, this study has sought to evaluate the impacts of the PFHI scheme in Chhattisgarh holistically. Chhattisgarh, which has a universal PFHI scheme and traditions of universalism in public policy, provides the opportune context for a comprehensive subnational analysis for assessing these impacts from an equity perspective.

The equity-based framework presented and applied in the study could be used for comprehensively assessing UHC and PFHI schemes elsewhere. This framework could also be used, as a heuristic device, by those interested in exploring other kinds of health programmes and schemes and holistically evaluating UHC-based reforms. This framework and its application contribute to the debate and discussions on PFHI schemes beyond enrolment to the determinants of real equity of access under such schemes. It also brings out the gaps in data and contributes to future research agenda. From the available evidence in Chhattisgarh, it is possible to draw a number of conclusions on the equity impacts of PFHI schemes in India.

Firstly, high enrolment rates and evidence of equitable enrolment (gender, social groups, economic and geography) from household surveys [105, 106] may mask specific pockets of inequity within households and among the most vulnerable communities [107, 119]. This highlights the need for routine population surveys to be complemented by in-depth, primary studies, examining experiences of specific vulnerable populations.

Secondly, and most importantly, equitable enrolment does not automatically lead to financial protection [105], to the acceptability of the PFHI scheme for the poor [105, 115] or to equity in utilisation [106]. The unequal availability of hospitals under the scheme was a key factor in unequal health service utilisation and resource distribution [106].

Thirdly, the public health sector continues to cater to the most vulnerable in Chhattisgarh, a finding that corroborates other studies in India [129]. Utilisation, especially in the private sector, was skewed towards the areas that had the least health and social need, exhibiting the ‘Inverse Care Law’ [130] with the resource allocation patterns deepening the ‘infrastructure inequality trap’ in the state [131]. High enrolment levels among vulnerable groups and in the most vulnerable districts effectively aided in mobilising funds into the scheme, which were effectively transferred from tribal and vulnerable to ‘better-off’ areas and from the public to the private sector, thus deepening inequity [60, 105, 106]. These findings raise questions regarding the effectiveness of private sector involvement in bringing about the equity goals of UHC, a concern that has also been raised by others [6, 90, 132–134].

Fourthly, technological innovations, such as the smart card and electronic information systems, do not, on their own, resolve problems rooted in wider normative and institutional failures. In Chhattisgarh, these include deeply entrenched practices of co-payment, dominant norms of healthcare as a market transaction rather than a right, poor governance, tolerance of conflicts of interests in decision-making and wider social inequalities [60, 115]. Mechanisms such as the biometric smart card, billing systems, and data reporting and sharing have not delivered on their promises nor led to greater accountability. More recent studies have indicated that these problems continue under the PMJAY [135]. Provider capture remains a central issue in the performance of PFHI-based policies to achieve UHC in the Indian context [136].

Finally, PFHI schemes exist within a political economy of health, which profoundly influences the implementation and everyday experience of these schemes. The development of PFHI schemes within the context of wider neoliberal policies promoting private sector provisioning has grave consequences for health equity and access [72].

Key gaps in knowledge relates to the impact of PFHI schemes on the health system. While there is some evidence on increasing budgetary allocations to PFHI schemes and the crowding out of funds to the public sector and other public health programmes [5], more research is needed on the impacts of this on public sector provision of services (including primary healthcare), human resource dynamics and supply chains. Research is specifically needed on the equity implications of changing resource allocations. In addition, shifting service delivery profiles through practices of ‘cherry picking’, the provision of narrow and selective services, and inducing demand [54, 61, 62] need to be better monitored.

The possibilities and constraints of technology and real-time data in improving transparency and accountability for the public merit further exploration. Research is needed into the systems and performance of government as the regulator and ‘steward’ of PFHI schemes, in monitoring implementation, ensuring that hospitals adhere to the contractual conditions and in promoting equity.

The study was of one state of India, Chhattisgarh, and therefore the findings have limitations in their generalisability to rest of India. There are, however, significant commonalities across the country – all states have a similar healthcare system, with a private/public mix, and with PFHI schemes primarily relying on private providers and focused on hospitalisation care. The findings on the impact of PFHI schemes are thus likely to be relevant to other states. Although the equity-based framework was designed to be comprehensive, the availability of data for different dimensions was variable.

## Conclusion

India has been championed as a prime example of advancing towards UHC through PFHI schemes. The stated intention of these PFHI schemes is to improve access to healthcare and provide financial protection to the vulnerable. However, as this study shows, the impact of RSBY/MSBY on equity of access and financial protection has been weak. Furthermore, to the extent that it has been studied, the implications for the public sector and of PFHI scheme funding predominantly channelled through the private sector are significant. At the point of service provision, the dominant normative and cultural orientation of healthcare as a commodity to be sold rather than a right remains unchallenged.

The findings of this study have immediate relevance to the present policy context in India, which is currently integrating existing PFHI schemes into a large expanded scheme, the PMJAY, for the whole country. The analysis has shown that an over-reliance on and rapid expansion of PFHI schemes in the Indian health system is unlikely to achieve UHC. Chhattisgarh is currently re-assessing the pitfalls of a private sector emphasis in its PFHI scheme and re-positioning the public health system at the core of service provision. Principles of solidarity, equity and rights are essential as the basis of health policy for universal healthcare. India still has some way to go in charting the pathways towards universal healthcare.

## Data Availability

The authors confirm that the data supporting the findings of this study are available within the article.

## Abbreviations

HPSR: Health policy and systems
LMICs: Low- and middle-income countries
MSBY: Mukhyamantri Swasthya Bima Yojana
OOP: Out-of-pocket
PDS: Public distribution system
PFHI: Publicly funded health insurance
PMJAY: Pradhan Mantri Jan Arogya Yojana
RSBY: Rashtriya Swasthya Bima Yojana
TPA: Third-party agency
UHC: Universal health coverage
